# Significant narrowing of the circumflex artery leads to worse outcomes than right coronary artery narrowing in patients with anterior myocardial infarction treated invasively

**DOI:** 10.1007/s12471-015-0678-5

**Published:** 2015-03-28

**Authors:** M. Kozuch, P. Kralisz, M. Rog-Makal, H. Bachorzewska-Gajewska, S. Dobrzycki

**Affiliations:** Department of Invasive Cardiology, Medical University of Bialystok, Sklodowskiej 24a street, 15-276 Bialystok, Poland

**Keywords:** Myocardial infarction, Revascularisation, Mortality, Prognosis

## Abstract

**Background:**

Occlusion of the circumflex artery (Cx) often does not present signs in the ECG. It can lead to delayed angiography during ST-elevation myocardial infarction (STEMI). The aim of this analysis was to determine if Cx narrowing is related to diverse outcomes in comparison with right coronary artery (RCA) stenosis in patients with STEMI, treated with percutaneous coronary intervention (PCI) of the left descending artery (LAD).

**Methods and results:**

Inclusion criteria were as follows: first STEMI treated with PCI of the LAD and additional significant (≥ 70 %) Cx or RCA narrowing—two-vessel disease. A total of 234 consecutive patients with STEMI were included. Total mortality was estimated during long-term follow-up, at mean 639 (± 224) days after STEMI. Patients with Cx narrowing constituted 46 % (*N* = 108) of the study population, and patients with RCA narrowing amounted to 54 % (*N* = 126). Patients with narrowing of the Cx had worse long-term outcomes in terms of mortality than patients with RCA narrowing (22 vs. 11 %, *p* < 0.05, respectively). Multiple regression analysis showed independent risk factors for death during long-term follow-up such as: age, ejection fraction and Cx narrowing.

**Conclusion:**

Significant Cx narrowing leads to worse outcomes than RCA narrowing in patients with STEMI treated with PCI of the LAD.

## Introduction

Over the past decades, the outcomes in patients with ST-elevation myocardial infarction (STEMI) have improved [1]. However, putting aside good early results, late outcomes are still not satisfactory, especially in patients with non-ST-elevation acute coronary syndromes in whom late prognosis is worse than in STEMI patients. Thus, searching for predictors of long-term mortality is still justified. Location of infarction is established as one such predictor. Inferior wall STEMI is thought to have a better long-term prognosis than anterior STEMI [2]. However, inferior STEMI may be the result of either circumflex artery (Cx) or right coronary artery (RCA) disease, leading to different outcomes. Nevertheless, significant narrowing of the Cx frequently does not present with ischemic signs on the ECG, even if the artery is occluded. Such a phenomenon can lead to the wrong qualification and delayed angiography during STEMI or stable coronary artery disease [3]. Hence, inadequate treatment of the narrowing of this artery can lead to worse outcomes. The number of arteries affected by atherosclerosis is an additional factor influencing the outcome. Moreover, prognosis may be affected by coronary artery dominance. It has been reported that left coronary artery dominance in the case of significant coronary artery disease is related to worse prognosis [4]. Moreover, the issue whether Cx or RCA disease is worse, still remains unclear. Additionally, different outcomes related to Cx or RCA stenosis in patients with anterior STEMI treated invasively have not been confirmed. Accordingly, the aim of this analysis was to establish if narrowing of the Cx is related to diverse outcomes in comparison with the narrowing of RCA in patients with STEMI treated with percutaneous coronary intervention (PCI) of the left anterior descending artery (LAD).

## Methods

The study was conducted among consecutive STEMI patients who underwent PCI of the LAD in our centre between 2005 and 2011. The study was based upon retrospective analysis of a clinical registry conducted in the authors’ own department. Inclusion criteria were as follows: first STEMI treated with PCI of LAD and additional, visually estimated, significant (≥ 70 %) narrowing of the Cx (segments 11–13) or RCA (segments 1–3)—two-vessel disease. Patients with ostial Cx stenosis or total occlusions of the Cx or RCA were excluded. Inclusion criteria were met by 234 patients who constituted a homogenous group. Every significant narrowing was treated invasively with planned, staged PCI. Staged PCI was performed within the first month after STEMI in patients with persisting clinical symptoms and/or a large vascularisation area of the narrowed vessel, according to the regimen prevailing in the centre. Patients with unsuccessful planned PCI were excluded from the analysis. Pharmacotherapy was administered according to the European Society of Cardiology guidelines binding at that time. All patients received a loading dose of clopidogrel (600 mg) and aspirin (300 mg). There were no differences concerning other pharmacological agents between the compared groups of patients. Mean follow-up was 639 (± 224) days. Total mortality was estimated at follow-up. Informed consent was obtained from each patient.

### Follow-up

Follow-up consisted of three stages. The first stage was a questionnaire sent to patients 2 years after myocardial infarction. The next stage, if the patient did not send the questionnaire back, was telephone contact with the patient or with his family. Missing data were collected from the Provincial Administration Office. Information concerning endpoint was obtained in 100 % of the cases. The office provides information concerning death of a person; however, the cause of death remains unknown. The study was approved by the local Bioethics Committee.

### Statistical analysis

Distribution of variables was assessed with the Kolmogorov–Smirnov test followed by the Student’s *t*-test, ANOVA test, or Mann–Whitney test for comparative analysis, the choice of a test depended on the distribution of a variable. The interdependences between constant variables were calculated with the Spearman or Pearson test depending on the statistical distribution. Correlations between dichotomous variables were analysed with the chi-square test. Multivariable analysis was performed with logistic regression testing. A *P* value < 0.05 was considered statistically significant. The results are presented as mean values with standard deviation or as percentage values presenting relative frequency. Statistical analysis was performed with Statistica 10.0 program (StatSoft, Inc. Tulsa, USA).

## Results

**Fig. 1:**
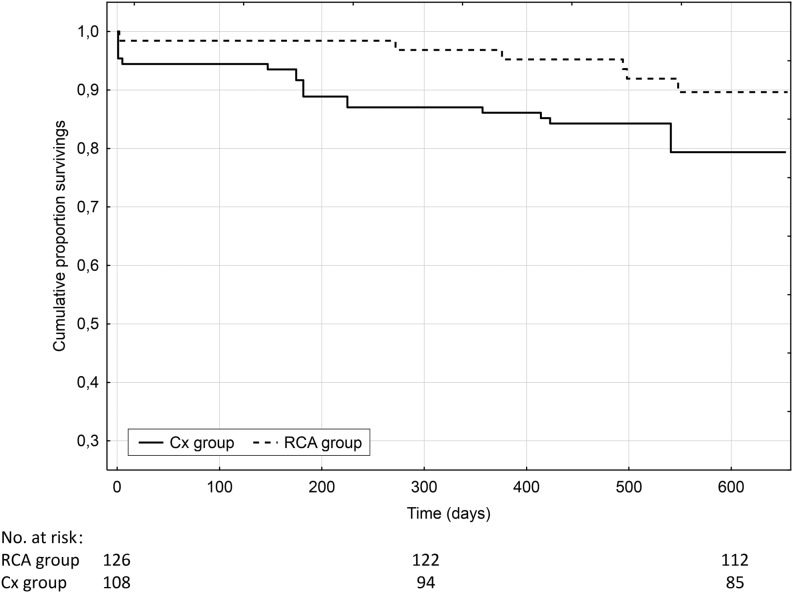
Kaplan–Meier curves displaying cumulative proportion survivals during long-term follow-up according to circumflex or right coronary artery narrowing (*p* < 0.05)

The study population was composed of 46 % (*N* = 108) of patients with Cx narrowing and 54 % (*N* = 126) with RCA narrowing. The Cx and RCA groups did not differ according to baseline clinical data (Table 1). There were no significant differences concerning either biochemical or history data (Table 2). Pharmacological treatment possibly affecting the outcome (beta-blockers, ACE inhibitors and statins) was also comparable at discharge. Long-term mortality was estimated at a level of 16 %. Mortality was higher in patients with Cx than with RCA narrowing (22 (*N* = 23) vs. 11 % (*N* = 14) respectively, *p* = 0.031, (Fig. 1). The ejection fraction of the left ventricle, age, glycaemia on admission, significant Cx stenosis and creatine kinase-myocardial band on admission were found to be risk factors for long-term mortality in univariate analysis (Table 3). Multiple regression analysis showed that independent risk factors for death during the follow-up were: age, ejection fraction and Cx narrowing (Table 4).

**Table 1:** Baseline clinical and angiographic characteristics

	RCA group (*N* = 126)		Cx group (*N* = 108)		*p*
	Mean or *N*	SD or %	Mean or *N*	SD or %	
Age (years)	64.92	9.92	66.30	12.44	ns
Male gender	91	72 %	74	69 %	ns
Body mass index (kg/m2)	28.10	5.45	26.65	3.09	ns
Systolic blood pressure on admission (mmHg)	153.71	145.54	146.60	36.11	ns
Diastolic blood pressure on admission (mmHg)	85.81	14.09	90.12	17.50	ns
Heart rate on admission (beats/minute)	75.23	16.27	78.80	16.86	ns
Ejection fraction of left ventricle (%)	40	10.44	42	10.32	ns
Mean Killip class on admission	1.42	0.51	1.46	0.54	ns
Time pain to door (minutes)	267.45	187.23	248.49	174.12	ns
Mean number of implanted stents	1.27	0.70	1.21	0.51	ns
POBA during index procedure	3	2 %	3	3 %	ns
% of implanted DES during index procedure	40	32 %	36	34 %	ns
POBA during second stage of PCI	0	0 %	1	1 %	ns
% of implanted DES during second stage of PCI	41	33 %	39	36 %	ns
PCI of proximal LAD during index procedure	52	41 %	42	39 %	ns
RCA dominance	82	65 %	72	67 %	ns

*RCA* right coronary artery, *Cx* circumflex artery, *ns* not significant, *LAD* left descending artery, *DES* drug-eluting stent, *POBA* plain old balloon angioplasty, *PCI* percutaneous coronary intervention, *SD* standard deviation

**Table 2:** Baseline biochemical and history characteristics

	RCA group (*N* = 126)		Cx group (*N* = 108)		*p*
	Mean or *N*	SD or %	Mean or *N*	SD or %	
Creatinine on admission (mg/dl)	1.01	0.22	1.06	0.19	ns
Total cholesterol on admission (mg %)	178.87	54.17	193.64	44.13	ns
HDL cholesterol on admission (mg %)	44.06	14.91	47.66	10.09	ns
LDL cholesterol on admission (mg %)	110.00	40.43	114.79	45.83	ns
Triglycerides on admission (mg %)	116.92	60.65	127.32	58.16	ns
Glucose level on admission (mg %)	119.38	33.72	115.50	43.92	ns
CKMB on admission (IU)	73.39	79.93	103.48	138.16	ns
Maximal CKMB (IU)	250.68	245.34	253.35	331.97	ns
Hypercholesterolaemia	59	47 %	54	50 %	ns
History of diabetes mellitus	25	20 %	20	19 %	ns
History of hypertension	73	58 %	62	57 %	ns

*RCA* right coronary artery, *Cx* circumflex artery, *ns* not significant, *HDL* high-density lipoprotein cholesterol, *LDL* low-density lipoprotein cholesterol, *CKMB* creatine kinase-myocardial band, *SD* standard deviation

**Table 3:** Significant risk factors for long-term mortality in univariate analysis

	Alive (*N* = 197)		Death (*N* = 37)		*p*
	Mean or *N*	SD or %	Mean or *N*	SD or %	
Ejection fraction of left ventricle (%)	42.88	9,37	26.55	6.97	< 0.001
Age (years)	64.14	10.60	72.80	8.24	< 0.001
Glucose level on admission (mg %)	112.89	29.68	150.25	61.52	0.005
Significant Cx stenosis	84	43 %	23	63 %	0.021
CKMB on admission	78.83	83.66	130.62	160.88	0.013

*Cx* circumflex artery, *CKMB* creatine kinase-myocardial band, *SD* standard deviation

**Table 4:** Risk factors of death during the follow-up in multiple regression analysis

	β	SE of β	B	SE of B	t(66)	*p*
Age (years)	0.2782	0.1024	0.0098	0.0036	2.7165	0.0080
CKMB on admission	0.1577	0.1070	0.0006	0.0004	1.4742	0.1442
Glucose level on admission (mg %)	0.0705	0.1045	0.0008	0.0012	0.6745	0.5018
Ejection fraction of left ventricle (%)	− 0.2777	0.1023	− 0.0119	0.0044	− 2.7144	0.0081
Significant Cx stenosis	0.2148	0.0948	0.1747	0,0771	2.2654	0.0261

*CKMB* creatine kinase-myocardial band, *Cx* circumflex artery, SE standard error

## Discussion

The long-term prognosis in patients with STEMI still requires improvement in the future. Different scales and factors predicting hospital and late outcomes have already been created [5–8]. Most of the scales used in mortality prediction lack angiographic and procedural aspects [9, 10]. One of the factors influencing the long-term efficacy of percutaneous interventions is the type of stent used for the procedure. Despite previous apprehension concerning drug-eluting stents (DES), they have turned out to be safe and efficient, also in the treatment of patients with acute coronary syndromes [11]. DES have significantly limited restenosis, which potentially may improve the prognosis of patients; however, data concerning the influence of restenosis on the mortality are equivocal [12, 13]. Nevertheless, it seems that the presence of coronary heart disease itself and its advancement are more important in the prognosis than the appearance of restenosis, which can be effectively treated today [13]. We did not evaluate the influence of the type of stent on mortality in our study. However, there were no differences concerning the prevalence of DES in the compared groups (Table 1).

It should be borne in mind that disease affecting different segments and arteries may lead to diverse outcomes. The location of a lesion in the LAD could be of key importance in the prognosis of patients with STEMI, due to the fact that the occlusion of the proximal LAD is related to more extensive heart muscle damage and therefore worse outcomes [10]. Nevertheless, the location of LAD occlusion did not present with differences in our analysis, which is also reflected in the lack of differentiation of the ejection fraction of the left ventricle, a direct exponent of the level of impairment of left ventricle. The Cx artery is the least frequent culprit vessel among patients treated invasively for STEMI [3]. Furthermore, patients with Cx occlusion are less likely to present ST-segment elevation, hence they remain underdiagnosed. Nevertheless, it has not been established if the outcome differs depending on whether the stenosis is in the Cx or RCA, in patients with anterior STEMI treated invasively. In the presented study, we found that patients with Cx narrowing present with worse outcomes. The explanation for such an observation has not been clearly defined. Patients with LAD narrowing usually have collateral circulation from the RCA artery. Thus, RCA narrowing in patients with anterior STEMI should lead to worse compensatory backflow to the occluded LAD, affecting the outcome more than Cx stenosis. Moreover, some studies suggested that Cx-related STEMI is usually smaller compared with RCA-related STEMI [14]. Such an observation should not be referred to the general population of patients with STEMI until coronary artery dominance is taken into consideration. Veltman et al. [15] reported that the prognosis of STEMI patients during a 30-day follow-up is worse in the case of left coronary artery dominance. According to the cited observation, discrepancies in the prognosis may result from the dominance of one artery and not directly from the type of the artery—Cx or RCA. In the presented study, the prevalence of right dominance was similar in the two groups, hence it may not constitute an explanation for the differences in mortality. On the other hand, the differences in mortality may stem from the fact that Cx stenosis in patients with anterior STEMI can be interpreted as an equivalent of left main disease, leading to worse outcome. Nevertheless, all the patients in our study underwent staged PCI procedures and were fully revascularised. This is of key importance taking into consideration the debate on the justification of complete revascularisation in STEMI. The latest publications have shown that patients who underwent complete coronary revascularisation in the acute phase of STEMI have a better prognosis than the others [16, 17]. These studies raise controversies, mainly due to the fact that the results are inconsistent with the results of large trials and meta-analyses [18–21]. Newly presented and vastly discussed trials seem to only support the justification of complete revascularisation in patients with myocardial infarction regarding the aspect of the improvement of the prognosis. However, they do not report the optimal time of complete revascularisation. Undoubtedly, we will be provided with more information from large randomised ongoing trials, e.g. the COMPLETE study. In the presented study, staged revascularisation was performed during a period of 1 month in all the subjects. Consequently, it should not affect the differences in mortality between the groups. Regardless of such treatment, the prognosis of patients with Cx stenosis was worse. Poor outcomes of the Cx group of patients may finally result from missing ECG changes at the time of Cx reocclusion during the observation. This suggests that the 12-lead ECG alone is often not enough for the diagnosis of patients with suspected Cx occlusion or narrowing. According to the authors, this may stem from the fact that electrocardiographically asymptomatic Cx reocclusion during the follow-up could be more common than in the case of RCA. The issue refers to long-term follow-up and not only to the anterior infarction-related period. This may result from atherosclerosis progression, acceleration of the narrowing in already diseased vessels and finally myocardial ischaemia. Additionally, it may be caused by acute occlusion related to the atherosclerotic plaque rupture in that artery. However, these are only unconfirmed assumptions. Therefore, we should be aware of features of Cx occlusion other than ST-segment elevation, such as isolated ST-segment depression in precordial leads (the greatest in leads V2 and V3) [22]. According to our findings, patients with anterior STEMI and Cx stenosis represent a group that requires a thorough evaluation during the follow-up period. Cx is a coronary artery requiring special attention because its stenosis in patients with anterior STEMI treated with PCI of the LAD leads to a worse prognosis in comparison with patients with RCA narrowing.

This study has a few limitations. First is the relatively small number of patients. However, to achieve a homogenous group of patients, we studied 3121 consecutive patients with STEMI. The observed correlation could be confirmed in the future with prospective and larger population studies. Moreover, we used overall mortality as an endpoint, due to the fact that it was not possible to define the cause of death in all cases. Thus, we are not certain whether all reported deaths were cardiovascular. Concluding, significant narrowing of the Cx leads to worse outcomes than narrowing of the RCA in patients with STEMI treated with PCI of the LAD. Thus, patients with Cx narrowing should be treated more cautiously and require special attention after anterior STEMI treated with PCI of LAD.

### Funding

This work was supported by grants 144-81532L and 133-81881L from Medical University of Bialystok.

### Conflict of interest

None.
